# Supplementary material for: Yeast Display Reveals Plentiful Mutations That Improve Fusion Peptide Vaccine-Elicited Antibodies Beyond 59% HIV-1 Neutralization Breadth
Source: Vaccines (Basel). 2025 Oct 27;13(11):1098. doi: 10.3390/vaccines13111098 (PMC12656643; doi:10.3390/vaccines13111098)
Supplement: Supplementary file 1 [file vaccines-13-01098-s001.zip › Figures S1-S9.pptx]

## Slide 1
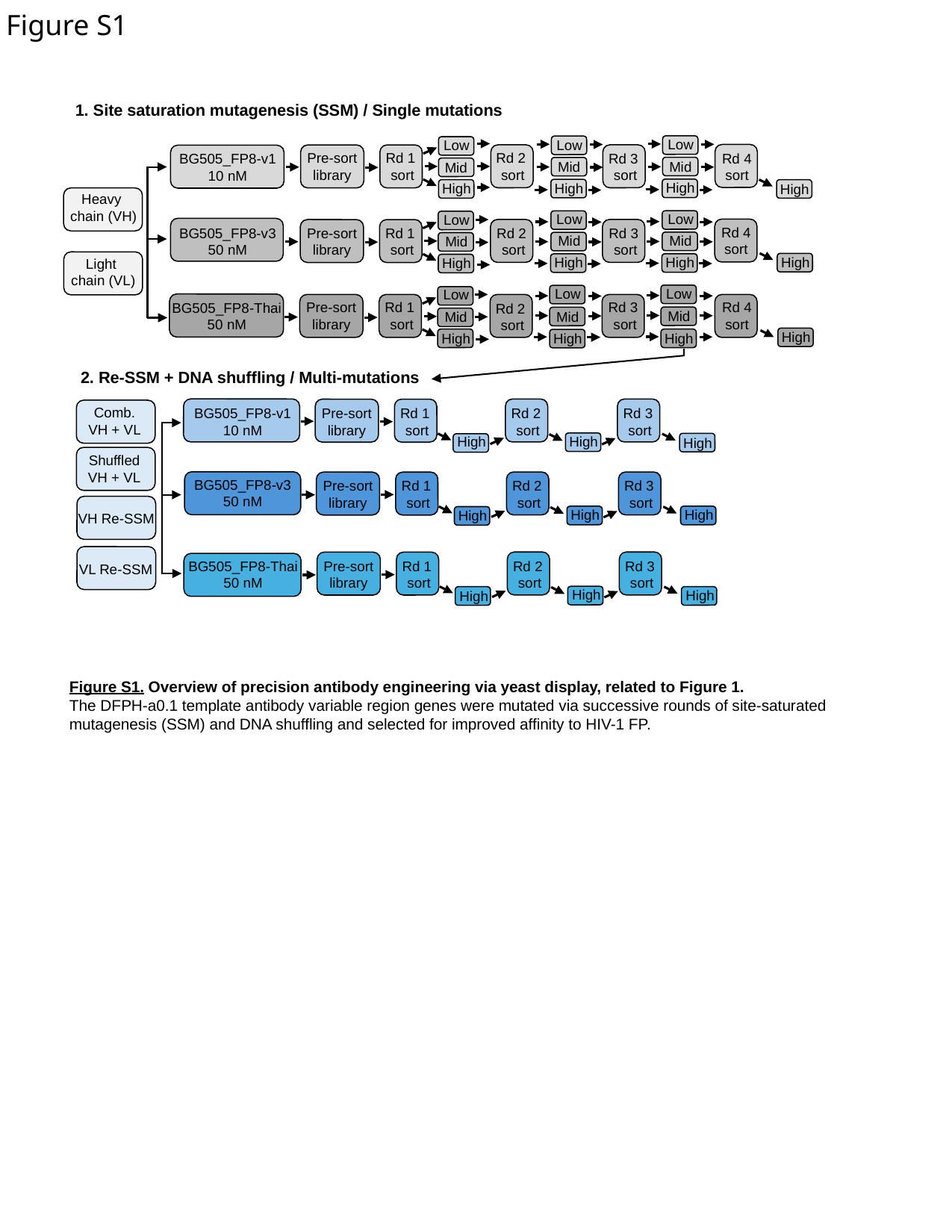

Figure S1
1. Site saturation mutagenesis (SSM) / Single mutations
Low
Low
Low
Pre-sort
library
Rd 1
sort
Rd 2
sort
Rd 4
sort
Rd 3
sort
BG505_FP8-v1
10 nM
Mid
Mid
Mid
High
High
High
High
Heavy
chain (VH)
Low
Low
Low
Rd 4
sort
Rd 3
sort
BG505_FP8-v3
50 nM
Pre-sort
library
Rd 1
sort
Rd 2
sort
Mid
Mid
Mid
High
High
High
High
Light
chain (VL)
Low
Low
Low
Pre-sort
library
Rd 1
sort
Rd 3
sort
Rd 4
sort
BG505_FP8-Thai
50 nM
Rd 2
sort
Mid
Mid
Mid
High
High
High
High
2. Re-SSM + DNA shuffling / Multi-mutations
Comb.
VH + VL
BG505_FP8-v1
10 nM
Pre-sort
library
Rd 1
sort
Rd 2
sort
Rd 3
sort
High
High
High
Shuffled
VH + VL
BG505_FP8-v3
50 nM
Pre-sort
library
Rd 1
sort
Rd 2
sort
Rd 3
sort
High
High
High
VH Re-SSM
BG505_FP8-Thai
50 nM
Pre-sort
library
Rd 1
sort
Rd 2
sort
Rd 3
sort
VL Re-SSM
High
High
High
Figure S1. Overview of precision antibody engineering via yeast display, related to Figure 1.
The DFPH-a0.1 template antibody variable region genes were mutated via successive rounds of site-saturated mutagenesis (SSM) and DNA shuffling and selected for improved affinity to HIV-1 FP.

## Slide 2
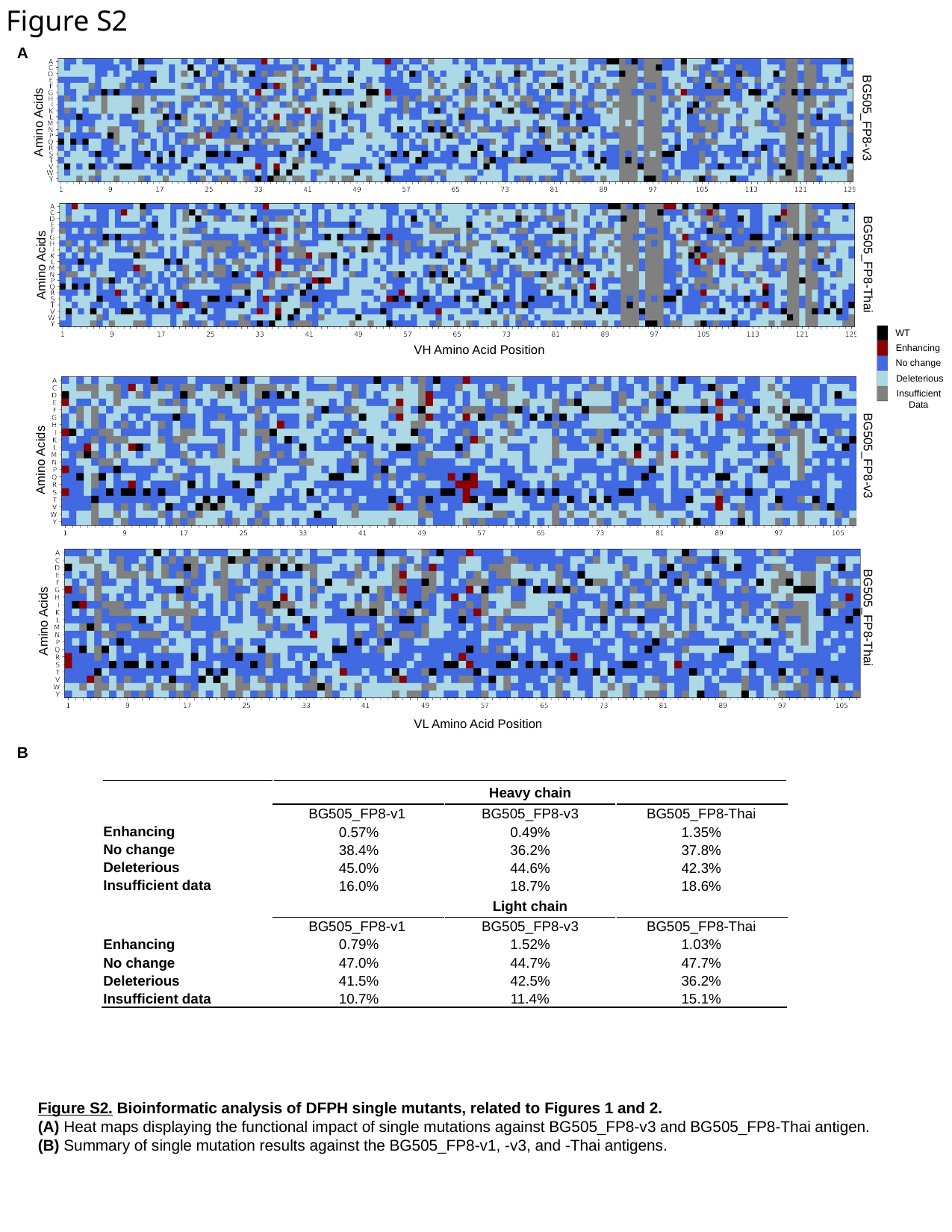

Figure S2
A
Amino Acids
BG505_FP8-v3
Amino Acids
BG505_FP8-Thai
WT
Enhancing
No change
Deleterious
Insufficient
Data
VH Amino Acid Position
Amino Acids
BG505_FP8-v3
Amino Acids
BG505_FP8-Thai
VL Amino Acid Position
B
| | | | | | |
| --- | --- | --- | --- | --- | --- |
| | | | | | |
| | | Heavy chain | | | |
| | | BG505\_FP8-v1 | BG505\_FP8-v3 | BG505\_FP8-Thai | |
| | Enhancing | 0.57% | 0.49% | 1.35% | |
| | No change | 38.4% | 36.2% | 37.8% | |
| | Deleterious | 45.0% | 44.6% | 42.3% | |
| | Insufficient data | 16.0% | 18.7% | 18.6% | |
| | | Light chain | | | |
| | | BG505\_FP8-v1 | BG505\_FP8-v3 | BG505\_FP8-Thai | |
| | Enhancing | 0.79% | 1.52% | 1.03% | |
| | No change | 47.0% | 44.7% | 47.7% | |
| | Deleterious | 41.5% | 42.5% | 36.2% | |
| | Insufficient data | 10.7% | 11.4% | 15.1% | |
| | | | | | |
| | | | | | |
| | | | | | |
| | | | | | |
Figure S2. Bioinformatic analysis of DFPH single mutants, related to Figures 1 and 2.
(A) Heat maps displaying the functional impact of single mutations against BG505_FP8-v3 and BG505_FP8-Thai antigen.
(B) Summary of single mutation results against the BG505_FP8-v1, -v3, and -Thai antigens.

## Slide 3
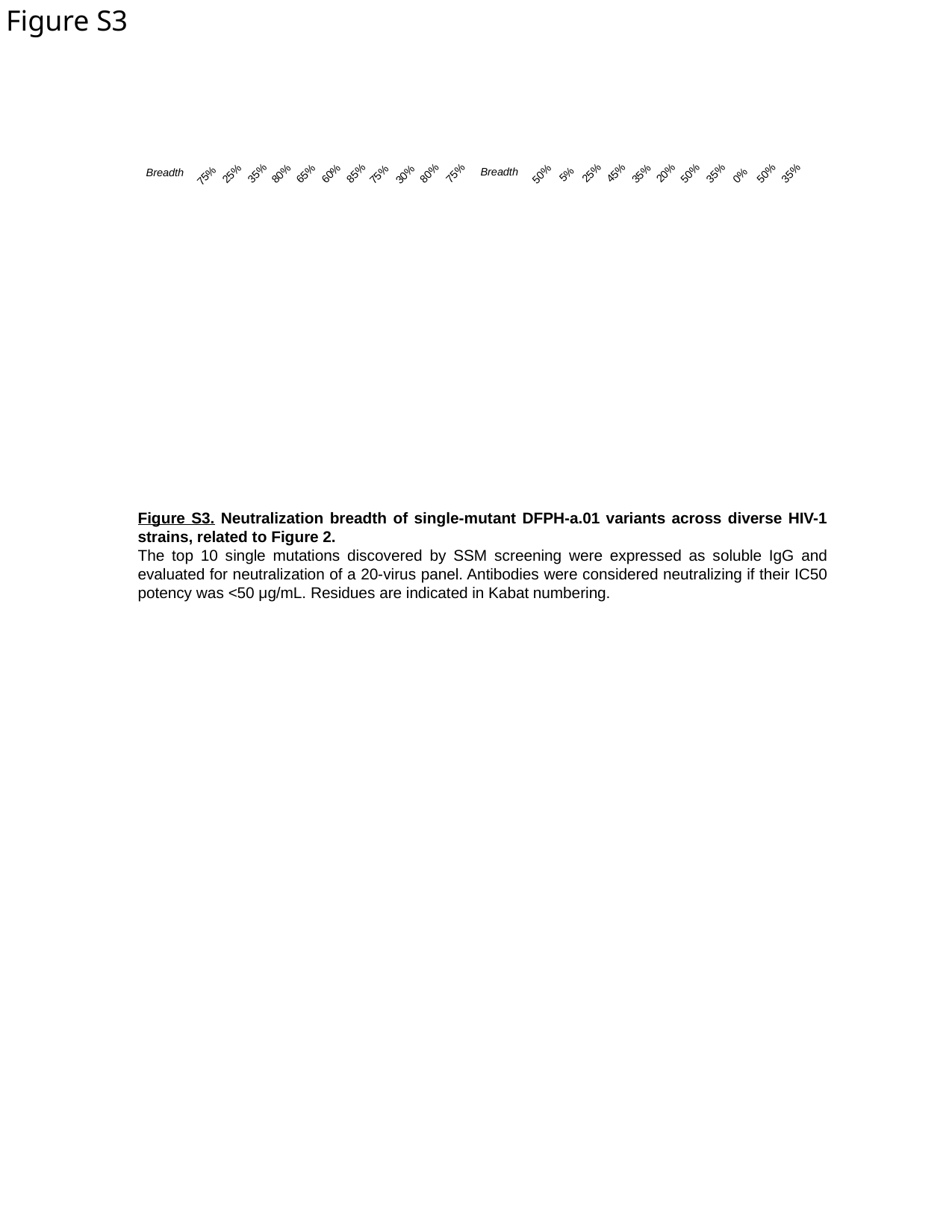

Figure S3
Breadth
Breadth
25%
45%
20%
50%
35%
50%
35%
35%
60%
85%
80%
75%
25%
80%
65%
35%
50%
5%
75%
30%
0%
75%
Figure S3. Neutralization breadth of single-mutant DFPH-a.01 variants across diverse HIV-1 strains, related to Figure 2.
The top 10 single mutations discovered by SSM screening were expressed as soluble IgG and evaluated for neutralization of a 20-virus panel. Antibodies were considered neutralizing if their IC50 potency was <50 μg/mL. Residues are indicated in Kabat numbering.

## Slide 4
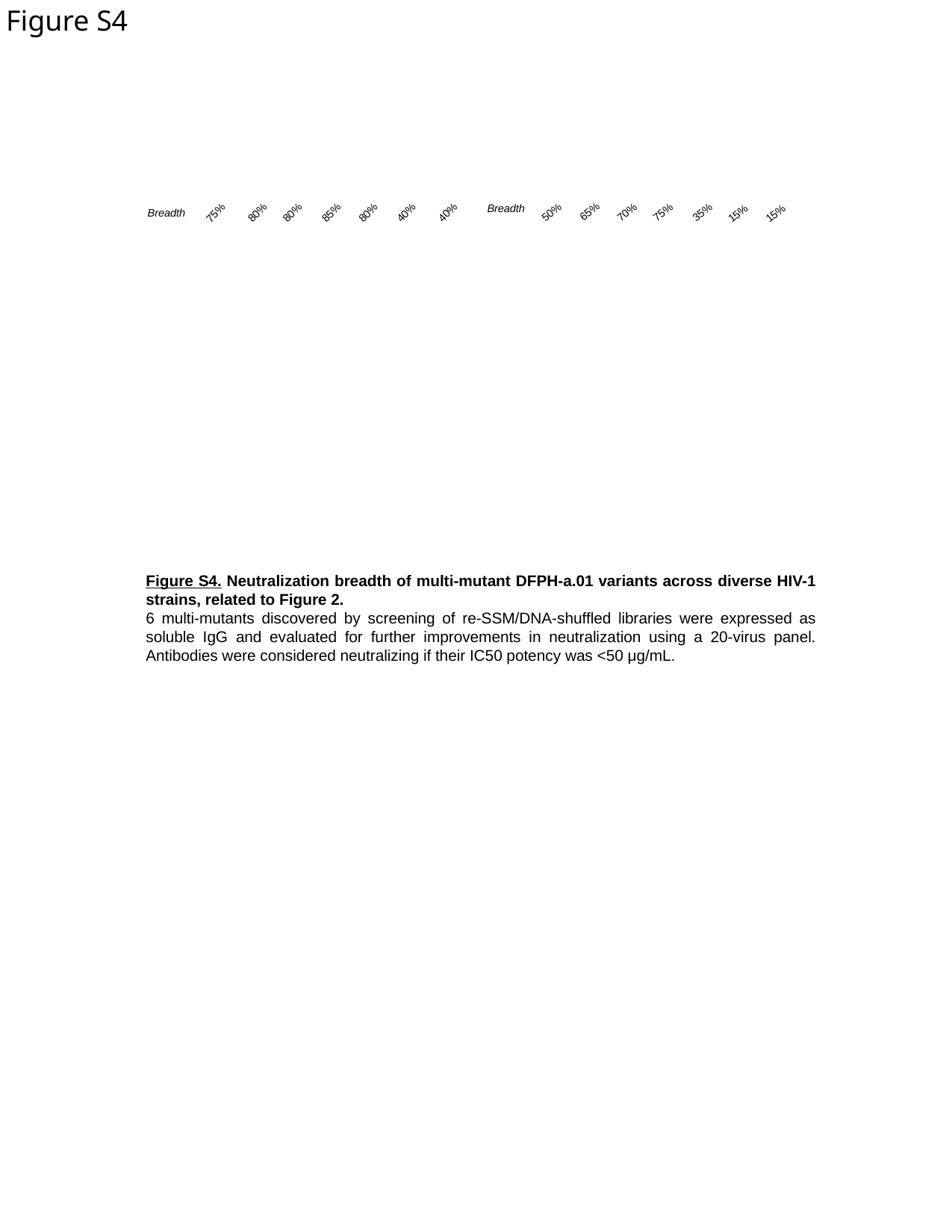

Figure S4
Breadth
65%
40%
50%
70%
75%
35%
40%
85%
80%
80%
80%
Breadth
75%
15%
15%
Figure S4. Neutralization breadth of multi-mutant DFPH-a.01 variants across diverse HIV-1 strains, related to Figure 2.
6 multi-mutants discovered by screening of re-SSM/DNA-shuffled libraries were expressed as soluble IgG and evaluated for further improvements in neutralization using a 20-virus panel. Antibodies were considered neutralizing if their IC50 potency was <50 μg/mL.

## Slide 5
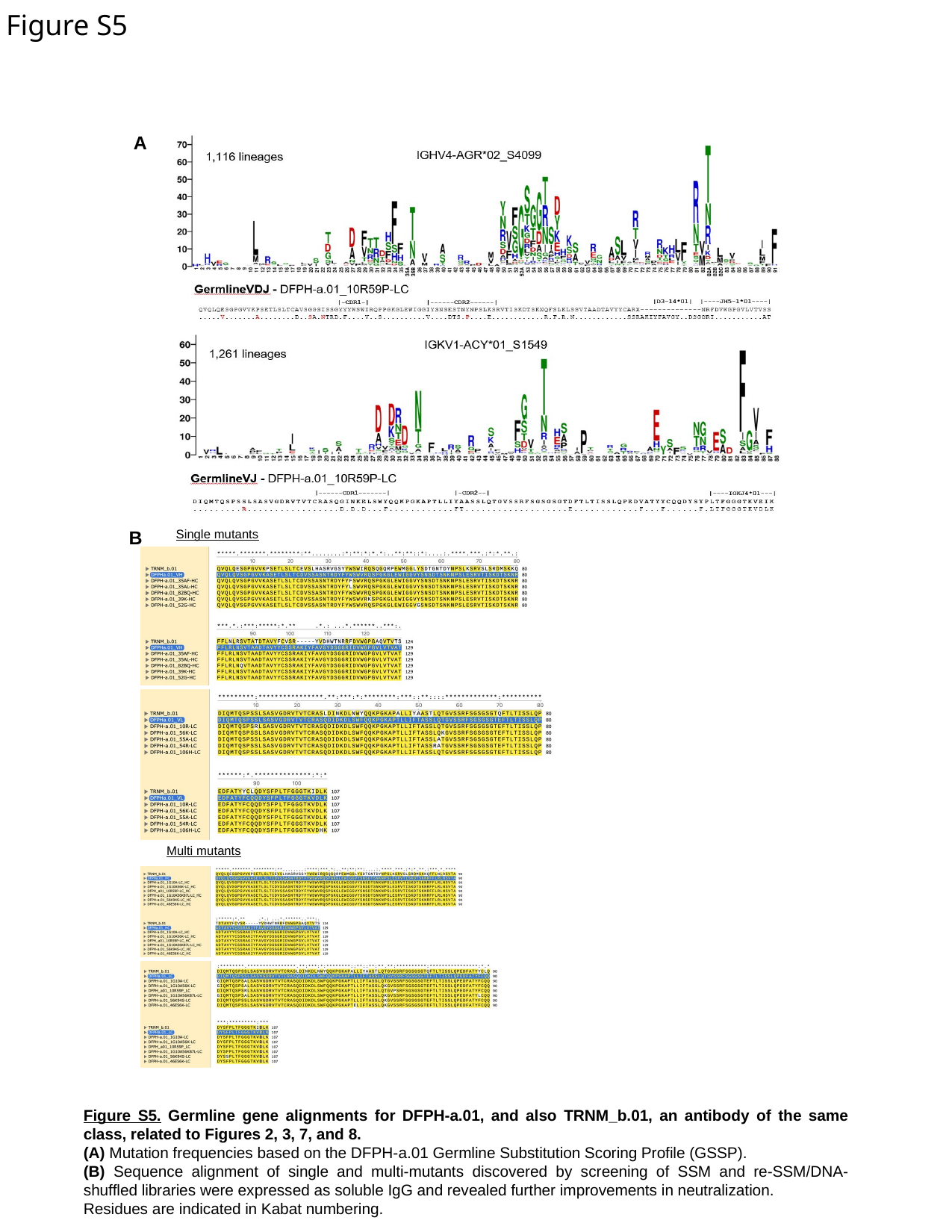

Figure S5
A
B
Single mutants
Multi mutants
Figure S5. Germline gene alignments for DFPH-a.01, and also TRNM_b.01, an antibody of the same class, related to Figures 2, 3, 7, and 8.
(A) Mutation frequencies based on the DFPH-a.01 Germline Substitution Scoring Profile (GSSP).
(B) Sequence alignment of single and multi-mutants discovered by screening of SSM and re-SSM/DNA-shuffled libraries were expressed as soluble IgG and revealed further improvements in neutralization.
Residues are indicated in Kabat numbering.

## Slide 6
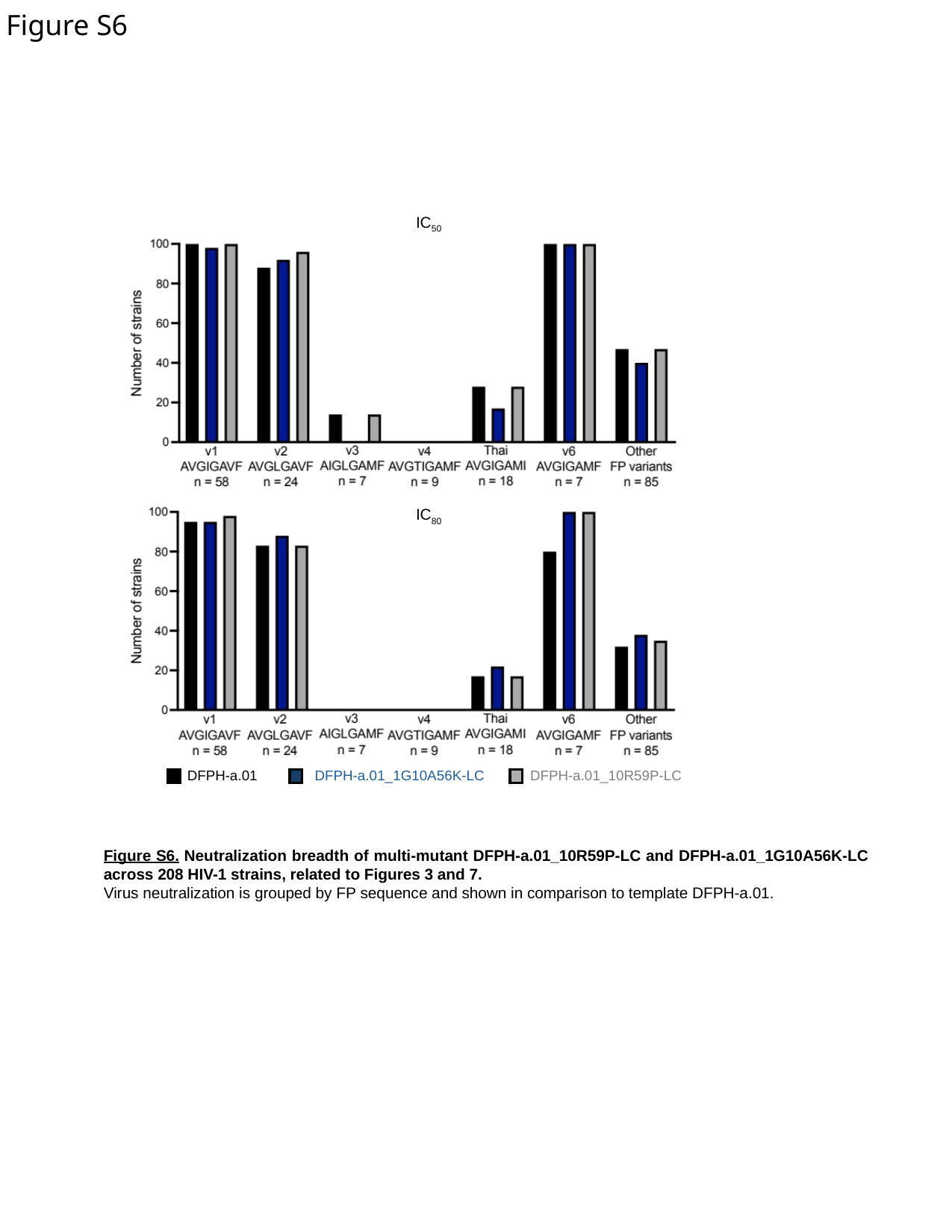

Figure S6
IC50
IC80
DFPH-a.01
DFPH-a.01_1G10A56K-LC
DFPH-a.01_10R59P-LC
Figure S6. Neutralization breadth of multi-mutant DFPH-a.01_10R59P-LC and DFPH-a.01_1G10A56K-LC across 208 HIV-1 strains, related to Figures 3 and 7.
Virus neutralization is grouped by FP sequence and shown in comparison to template DFPH-a.01.

## Slide 7
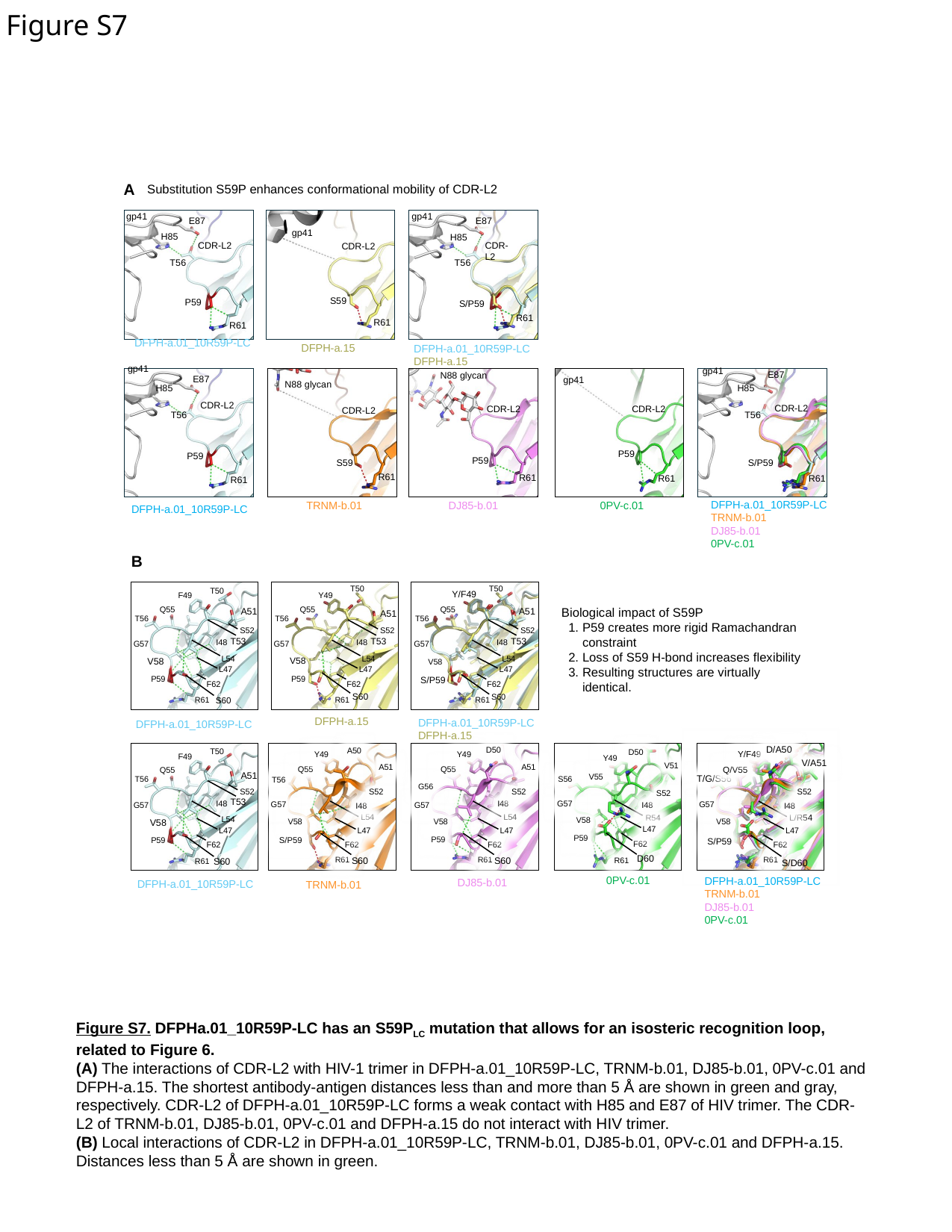

Figure S7
A
Substitution S59P enhances conformational mobility of CDR-L2
gp41
E87
H85
CDR-L2
T56
P59
R61
DFPH-a.01_10R59P-LC
gp41
CDR-L2
S59
R61
DFPH-a.15
gp41
E87
H85
CDR-L2
T56
S/P59
R61
DFPH-a.01_10R59P-LC
DFPH-a.15
gp41
E87
H85
CDR-L2
T56
P59
R61
DFPH-a.01_10R59P-LC
N88 glycan
CDR-L2
S59
R61
TRNM-b.01
N88 glycan
CDR-L2
P59
R61
DJ85-b.01
gp41
CDR-L2
P59
R61
0PV-c.01
gp41
E87
H85
CDR-L2
T56
S/P59
R61
DFPH-a.01_10R59P-LC
TRNM-b.01
DJ85-b.01
0PV-c.01
B
T50
F49
Q55
A51
T56
S52
T53
I48
G57
L54
V58
L47
P59
F62
S60
R61
DFPH-a.01_10R59P-LC
T50
Y49
Q55
A51
T56
S52
T53
I48
G57
L54
V58
L47
P59
F62
S60
R61
DFPH-a.15
T50
Y/F49
Q55
A51
T56
S52
T53
I48
G57
L54
V58
L47
S/P59
F62
S60
R61
DFPH-a.01_10R59P-LC
DFPH-a.15
T50
F49
Q55
A51
T56
S52
T53
I48
G57
L54
V58
L47
P59
F62
S60
R61
DFPH-a.01_10R59P-LC
A50
Y49
A51
Q55
T56
S52
G57
I48
L54
V58
L47
S/P59
F62
S60
R61
TRNM-b.01
D50
Y49
A51
Q55
G56
S52
I48
G57
L54
V58
L47
P59
F62
S60
R61
DJ85-b.01
D50
Y49
V51
V55
S56
S52
G57
I48
R54
V58
L47
P59
F62
D60
R61
0PV-c.01
D/A50
Y/F49
V/A51
Q/V55
T/G/S56
S52
G57
I48
L/R54
V58
L47
S/P59
F62
R61
S/D60
DFPH-a.01_10R59P-LC
TRNM-b.01
DJ85-b.01
0PV-c.01
Biological impact of S59P
P59 creates more rigid Ramachandran constraint
Loss of S59 H-bond increases flexibility
Resulting structures are virtually identical.
Figure S7. DFPHa.01_10R59P-LC has an S59PLC mutation that allows for an isosteric recognition loop, related to Figure 6.
(A) The interactions of CDR-L2 with HIV-1 trimer in DFPH-a.01_10R59P-LC, TRNM-b.01, DJ85-b.01, 0PV-c.01 and DFPH-a.15. The shortest antibody-antigen distances less than and more than 5 Å are shown in green and gray, respectively. CDR-L2 of DFPH-a.01_10R59P-LC forms a weak contact with H85 and E87 of HIV trimer. The CDR-L2 of TRNM-b.01, DJ85-b.01, 0PV-c.01 and DFPH-a.15 do not interact with HIV trimer.
(B) Local interactions of CDR-L2 in DFPH-a.01_10R59P-LC, TRNM-b.01, DJ85-b.01, 0PV-c.01 and DFPH-a.15. Distances less than 5 Å are shown in green.

## Slide 8
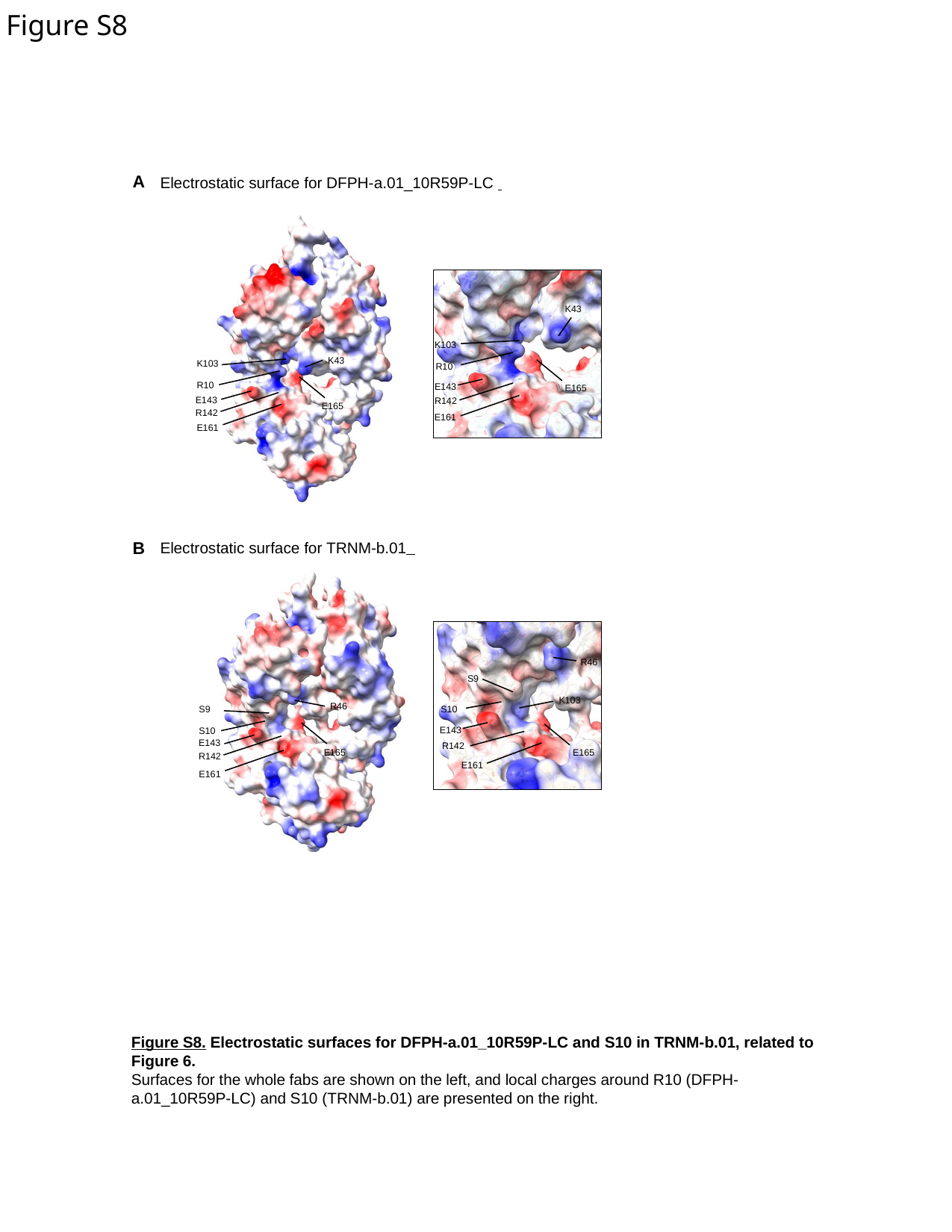

Figure S8
A
Electrostatic surface for DFPH-a.01_10R59P-LC
K43
K103
R10
E143
E165
R142
E161
K43
K103
R10
E143
E165
R142
E161
B
Electrostatic surface for TRNM-b.01
R46
S9
S10
E143
E165
R142
E161
R46
S9
K103
S10
E143
R142
E165
E161
Figure S8. Electrostatic surfaces for DFPH-a.01_10R59P-LC and S10 in TRNM-b.01, related to Figure 6.
Surfaces for the whole fabs are shown on the left, and local charges around R10 (DFPH-a.01_10R59P-LC) and S10 (TRNM-b.01) are presented on the right.

## Slide 9
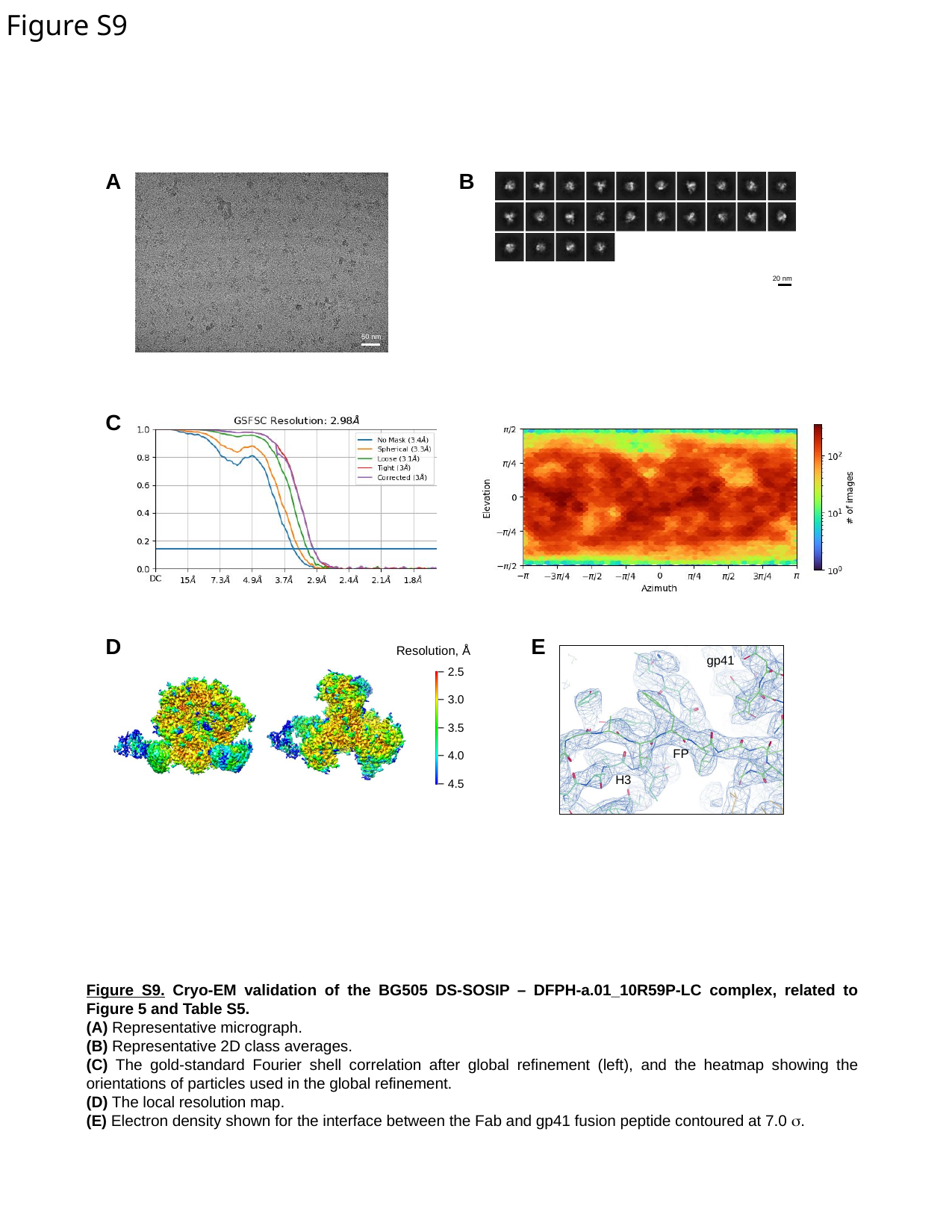

Figure S9
A
B
20 nm
50 nm
C
D
E
Resolution, Å
− 2.5
− 3.0
− 3.5
− 4.0
− 4.5
gp41
FP
H3
Figure S9. Cryo-EM validation of the BG505 DS-SOSIP – DFPH-a.01_10R59P-LC complex, related to Figure 5 and Table S5.
(A) Representative micrograph.
(B) Representative 2D class averages.
(C) The gold-standard Fourier shell correlation after global refinement (left), and the heatmap showing the orientations of particles used in the global refinement.
(D) The local resolution map.
(E) Electron density shown for the interface between the Fab and gp41 fusion peptide contoured at 7.0 .
