# Supplementary material for: Yeast Display Reveals Plentiful Mutations That Improve Fusion Peptide Vaccine-Elicited Antibodies Beyond 59% HIV-1 Neutralization Breadth
Source: Vaccines (Basel). 2025 Oct 27;13(11):1098. doi: 10.3390/vaccines13111098 (PMC12656643; doi:10.3390/vaccines13111098)
Supplement: Supplementary file 1 [file vaccines-13-01098-s001.zip › Table S5.pdf]

Table S5. Cryo-EM data collection, refinement, and validation statistics for BG505 DS-SOSIP – DFPH-a.01_10R59P-LC Fab complex, related to Figure 5 and Figure S8.

	BG505 DS-SOSIP<br>DFPH-a.01_10R59P-LC<br>(EMD-72108)<br>(PDB: 9Q0W)
**Data collection and processing**	
Magnification	105,000
Voltage (kV)	300
Electron exposure (e–/Å^2)	43.5
Defocus range (μm)	-0.75 to -2.0
Pixel size (Å)	0.415 (0.83)
Symmetry imposed	C1
Initial particle images (no.)	852,240
Final particle images (no.)	347,691
Map resolution (Å)	3.0
FSC threshold	0.143
**Refinement**	
Initial model used (PDB code)	8EUV
Model resolution (Å)	3.0
FSC threshold	0.143
Model composition	
Non-hydrogen atoms	24,735
Protein residues	3,042
Ligands	87
B factors (Å^2)	
Protein	88.5
Ligand	74.8
R.m.s. deviations	
Bond lengths (Å)	0.003
Bond angles (°)	0.632
Validation	
MolProbity score	1.09
Clashscore	1.90
Poor rotamers (%)	1.08
Ramachandran plot	
Favored (%)	97.46
Allowed (%)	2.44
Disallowed (%)	0.10
