# Supplementary material for: Yeast Display Reveals Plentiful Mutations That Improve Fusion Peptide Vaccine-Elicited Antibodies Beyond 59% HIV-1 Neutralization Breadth
Source: Vaccines (Basel). 2025 Oct 27;13(11):1098. doi: 10.3390/vaccines13111098 (PMC12656643; doi:10.3390/vaccines13111098)
Supplement: Supplementary file 1 [file vaccines-13-01098-s001.zip › Table S6.pdf]

Table S6. BG505 DS-SOSIP (HIV4571) - DFPH-a.01_10R59P-LC Fab interface details, related to Figure 8.

Residue	Bond	Epitope BSA, Å^2	Residue	Bond	Paratope BSA, Å^2
**gp41 chain B**			**Heavy Chain**		
B:ALA 512		23.77 ||	G:SER 28		6.24 ||
B:VAL 513		6.13 |	G:THR 30		17.96 ||
B:GLY 514	H	66.39 |||||||	G:ARG 31		20.06 |
B:ILE 515	H	108.29 |||||||	G:ASP 32	H	54.72 ||||||
B:GLY 516		55.20 |||||||||	G:TYR 33	H	53.49 ||||
B:ALA 517	H	87.48 |||||||||	G:PHE 34		11.26 ||||
B:VAL 518	H	116.91 ||||||||	G:TYR 35		25.66 ||||||
B:PHE 519	H	136.65 |||||||	G:TYR 52		29.31 ||||
B:LEU 520	H	65.66 |||||	G:ARG 95	H	37.08 ||||
B:GLY 521		6.36 ||	G:ALA 96		25.64 |||||||||
B:ALA 525		3.68 ||||	G:LYS 97	H	51.95 ||||||
B:SER 528		13.46 ||||	G:ILE 98		46.52 |||||
B:ALA 532		28.82 |||||	G:TYR 99	H	69.84 ||||||
B:MET 535		84.94 |||||	G:PHE 100		138.40 ||||||||
B:THR 536		19.74 ||||||||	G:ALA 100A		31.86 |||||
			G:VAL 100B		21.98 ||
			G:TYR 100D		43.03 |||
			G:SER 100F		3.35 |
			G:ARG 100I		32.90 |||
			G:ILE 100J		4.62 |
**gp120 chain A**			**Heavy Chain**		
A:ASN 80		57.97 |||||	G:GLN 1		14.68 |
A:PRO 81		9.09 ||	G:SER 25		12.26 ||
A:GLN 82		61.81 |||||	G:SER 26		4.35 |
A:GLU 83		5.96 |	G:ALA 27		70.91 ||||||||
			G:SER 28		18.73 ||||
			G:ASN 29		1.77 |
**gp41 chain B**			**Light Chain**		
B:ALA 512	HS	55.63 |||||	H:ASP 32	HS	29.55 |||||
B:VAL 513	H	87.07 |||||	H:ASP 91	H	36.96 |||||
B:GLY 514		18.77 |||	H:TYR 92		19.75 ||
B:ILE 515		4.19 |	H:SER 93		7.55 ||
			H:PHE 94		40.78 |||
			H:LEU 96		8.95 ||
**gp120 chain A**			**Light Chain**		
A:HIS 85		27.89 ||||	H:THR 56		54.23 |||||
A:GLU 87		18.18 ||			
**gp41 chain F**			**Heavy Chain**		
F:GLN 640		2.87 |	G:ARG 31	HS	98.15 |||||
F:ILE 641		33.94 ||||	G:ALA 100A		5.16 |
F:GLY 644		19.78 |||||	G:VAL 100B		32.91 |||
F:LEU 645		11.08 |||	G:TYR 100D	H	43.33 |||
F:GLU 648	HS	51.79 |||||			
F:GLN 652	H	33.13 ||||			
F:LYS 655		12.56 ||			
**gp120 glycan**			**Heavy Chain**		
a:NAG 691		43.96 ||	G:PHE 100		16.88 ||
			G:SER 100F		23.34 ||||||
a:NAG 692		2.08 |	G:SER 100F		2.21 |

https://www.ebi.ac.uk/pdbe/pisa/

BSA Buried Surface Area, Å²
HSDC Residues making **H**ydrogen/**D**isulphide bond, **S**alt bridge or **C**ovalent link
|||| Buried area percentage, one bar per 10%
